# An integrative, translational approach to understanding rare and orphan genetically based diseases

**DOI:** 10.1098/rsfs.2012.0055

**Published:** 2013-04-06

**Authors:** Robert Hoehndorf, Paul N. Schofield, Georgios V. Gkoutos

**Affiliations:** 1 Department of Physiology, Development and Neuroscience, University of Cambridge, Downing Street, Cambridge CB2 3EG, UK; 2 Department of Computer Science, University of Aberystwyth, Old College, King Street, Aberystwyth SY23 2AX, UK

**Keywords:** phenotype, animal model, rare disease, orphan disease, Orphanet, biomedical informatics

## Abstract

PhenomeNet is an approach for integrating phenotypes across species and identifying candidate genes for genetic diseases based on the similarity between a disease and animal model phenotypes. In contrast to ‘guilt-by-association’ approaches, PhenomeNet relies exclusively on the comparison of phenotypes to suggest candidate genes, and can, therefore, be applied to study the molecular basis of rare and orphan diseases for which the molecular basis is unknown. In addition to disease phenotypes from the Online Mendelian Inheritance in Man (OMIM) database, we have now integrated the clinical signs from Orphanet into PhenomeNet. We demonstrate that our approach can efficiently identify known candidate genes for genetic diseases in Orphanet and OMIM. Furthermore, we find evidence that mutations in the *HIP1* gene might cause Bassoe syndrome, a rare disorder with unknown genetic aetiology. Our results demonstrate that integration and computational analysis of human disease and animal model phenotypes using PhenomeNet has the potential to reveal novel insights into the pathobiology underlying genetic diseases.

## 1. Introduction

Two major goals of biomedical research are the in-depth understanding of the function of genes and their role in human disease. To achieve these goals, research in genetics seeks to understand the functions of individual genes, their interactions with other genes, the molecular consequences of allelic variation and how this variation interacts with environmental factors. In order to study these parameters, researchers use a variety of organisms and approaches, such as forward and reverse genetics, in an attempt to link the phenotypic manifestations observed in an organism with their genetic basis.

In addition to hypothesis-based studies, systematic mutagenesis and phenotyping programmes are now being implemented for several model organisms, with the aim of describing the phenotypes associated with mutations in every protein-coding gene, revealing the genes' functions, the structure and dynamics of physiological pathways as well as providing insights into the pathobiology of disease. While the manifestations of mutations in homologous genes might be expected to give rather diverse phenotypes in different organisms, it has been shown that in many cases, particularly between vertebrates, phenotypes are remarkably conserved, implying that the underlying physiological pathways in which these genes function are themselves highly conserved. As such, animal models are a valuable tool for the investigation of gene function and the study of human disease.

One of the main challenges is to compare phenotypes systematically across species and to translate the insights from animal model research into an understanding of human traits and disease. Achieving this goal would allow us to capture variation and link biological processes through to phenotypes, enabling us to increase the speed by which findings from basic animal research are translated into clinical applications that benefit human health and increase our understanding of basic biological processes. In the context of clinical research, the Online Mendelian Inheritance in Man (OMIM) knowledgebase [1], a database that catalogues the association between human phenotypes and their causative genes, and the Orphanet database [2], a database dedicated to information on rare diseases and orphan drugs, form two of the main information sources for phenotypic manifestations associated with human genetic disease.

To characterize phenotypes, model organism databases and disease information sources use controlled vocabularies, or ontologies, to provide standardized descriptions of phenotype observations. Ontologies in biology provide structured, controlled vocabularies of terms that can be used to annotate complex datasets [3], and a large number of phenotype ontologies have been developed in the context of clinical and biomedical research as well as for the annotation of mutant animal model phenotypes. Table 1 lists some of the major phenotype ontologies that are currently in use.

**Table 1.** Overview over phenotype vocabularies and ontologies. OMIM, Online Mendelian Inheritance in Man; MGI, Mouse Genome Informatics; RGD, Rat Genome Database; SGD, *Saccharomyces* Genome Database.

ontology/vocabulary	species/domain	resources
Human Phenotype Ontology (HPO) [4]	human, clinical phenotypes	OMIM [1]
Orphanet signs and symptoms	human, clinical phenotypes	Orphanet [2]
Mammalian Phenotype Ontology (MP) [5]	mammals, primarily mouse	MGI [6], RGD [7]
FlyBase Controlled Vocabulary	*Drosophilidae*	FlyBase [8]
DictyBase Phenotype Ontology	*Dictyostelium discoideum*	DictyBase [9]
Ascomycete Phenotype Ontology	*Saccharomyces*	SGD [10]
*Caenorhabditis elegans* Phenotype Ontology [11]	*Caenorhabditis elegans*	WormBase [12]
Fission Yeast Phenotype Ontology	*Schizosaccharomyces pombe*	PomBase [13]
Plant Trait Ontology [14,15]	flowering plants	Gramene Resource for Comparative Grass Genomics [16], The *Arabidopsis* Information Resource [17]

In order to integrate phenotypes across species, the Phenotype And Trait Ontology (PATO) was created as the key to a framework that allows the description and integration of quantitative and qualitative phenotype-related information across different levels of granularity (i.e. across scales reaching from the molecular level over the organizational levels of the organelle, cell, tissue and organ to the whole organism), different domains and species [18]. PATO allows for the description of phenotypes by combining qualities (such as colours, sizes, masses, lengths) with the entities of which they are a quality. These entities are either anatomical structures (represented in anatomy ontologies), biological processes, functions or cellular components (represented in the Gene Ontology (GO), and other biological entities (described, e.g. in the CellType Ontology). This allows PATO-based phenotype descriptions to be integrated across species, and several thousand PATO-based definitions of phenotype terms in major phenotype ontologies have already been created [19].

Recently, we have used these definitions to develop PhenomeNet, a phenotype-based system to prioritize candidate genes for diseases based on comparing the similarity between animal model phenotypes and human disease phenotypes [20]. PhenomeNet integrates phenotype vocabularies of multiple model organism species, and systematically compares the similarity of experimentally derived phenotypes from mutagenesis experiments with human disease phenotypes. PhenomeNet then computes the pairwise similarity for all included phenotypes (either from animal models or descriptions of diseases) and suggests candidate disease models based on phenotypic similarity. In contrast to ‘guilt-by-association’ approaches, the PATO-based integration of phenotypes enables the *direct* comparison of phenotypes in different species (such as human and mouse) and can, therefore, be applied to suggest candidate genes for rare and orphan diseases for which the molecular basis is not known.

We have now extended the PhenomeNet approach by integrating the clinical signs associated with disorders from Orphanet [2]. We quantitatively evaluate the success of PhenomeNet for prioritizing candidate genes based on Orphanet's clinical signs using an analysis of the receiver operating characteristic (ROC) curve [21], and use our method for identifying candidate genes for diseases whose aetiology is unknown. Based on the similarity between phenotypic manifestations observed in mutant mice and the clinical signs associated with disorders in Orphanet, we present and discuss evidence that the *HIP1* gene may be responsible for Bassoe syndrome.

Our results demonstrate that integration and computational analysis of human disease and animal model phenotypes using PhenomeNet has the potential to reveal novel insights into the pathobiology underlying genetic diseases. All our results and a web-based interface that can be used to query and explore our PhenomeNet system can be found at http://phenomebrowser.net.

## 2. Results and discussion

### 2.1. Performance of Orphanet-based disease gene discovery

We have now incorporated the Orphanet phenotypes into PhenomeNet, and use PhenomeNet to perform a pairwise comparison of the phenotypic similarity to all other included phenotypes, assuming that phenotypic similarity is indicative of an underlying biological relation. To evaluate our integration results for Orphanet, we compare PhenomeNet's rankings against known gene–disease associations taken from the Mouse Genome Informatics (MGI) database [6], against OMIM's gene–disease associations and against Orphanet's gene–disease associations. MGI's gene–disease associations are based on OMIM, i.e. they associate mouse models with OMIM disease identifiers, but manually evaluate assertions in publications making this a gold-standard resource [22]. To evaluate against OMIM, we map the Orphanet disease identifiers to their corresponding OMIM identifier using the mappings provided by Orphanet. Because not all OMIM diseases can be mapped to Orphanet diseases, we only perform this mapping in one direction. Orphanet associates human genes with diseases, and we use the human–mouse orthology associations provided by the MGI to map humans genes to their mouse equivalent.

**Figure 1.**
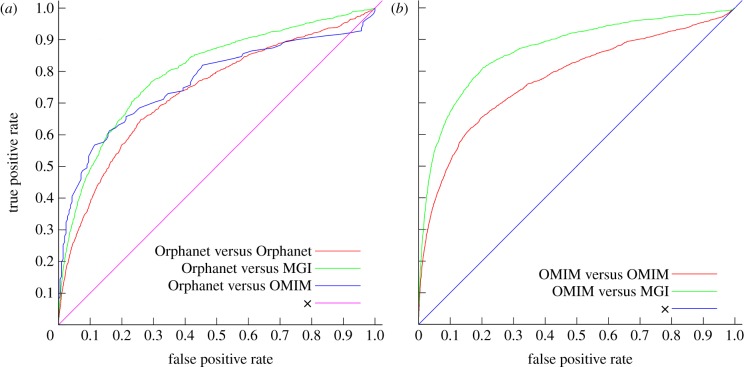
The figure shows the ROC curves for predicting disease genes based on phenotypic similarity in the PhenomeNet system. A ROC curve is a plot of the true positive rate of a classifier as a function of its false positive rate. Here, we rank animal model phenotypes based on their phenotypic similarity to a disease phenotype, and evaluate true and false positives rates for each rank (starting with the most similar animal model phenotypes for a disease phenotype). The true positive rate is calculated as the fraction of known gene–disease associations identified (on the *y*-axis), and the false positive rate is the fraction of gene–disease pairs identified in which the gene is *not known* to be involved in the disease (on the *x*-axis). The ROC AUC is a quantitative measure of the success of predicting disease genes through comparisons of phenotypes. A ROC AUC of 0.5 indicates a random classifier (i.e. the true positive rate increases proportional to the false positive rate), a ROC AUC above 0.5 indicates that the prediction is better than random, and a ROC AUC of 1 would indicate a perfect classifier. (*a*) The ROC curves resulting from comparing Orphanet disease phenotypes with mouse model phenotypes and compared with known gene–disease associations from Orphanet (AUC 0.734), OMIM (AUC 0.764) and MGI (AUC 0.798). (*b*) The ROC curves resulting from comparing OMIM disease phenotypes with mouse model phenotypes and comparing against known gene–disease associations from OMIM (AUC 0.777) and MGI (AUC 0.868). (Online version in colour.)

To validate our approach for identifying gene–disease associations, we use ROC analysis [21]. A ROC curve is a plot of the true positive rate of a classifier as a function of its false positive rate. The area under the ROC curve (ROC AUC) is a quantitative measure of the classifier's performance. To compute the true and false positive rates, we first identify, for each disease, the genes that have been identified as being involved in the disease in Orphanet, OMIM or MGI. We treat these gene–disease pairs as positive instances. In the absence of a large set of negative gene–disease associations, we treat all other associations as negative instances for the purpose of our evaluation. As second step, we rank animal model phenotypes based on their similarity to a disease phenotype, and iterate through the ranks starting with the most similar animal model phenotype. At each rank *r*, we compute the true positive rate TPR(*r*) as2.1
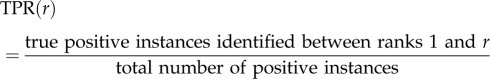
and the false positive rate FPR(*r*) as2.2
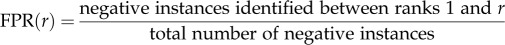
Using Orphanet's gene–disease associations as positive instances, the resulting ROC AUC of our approach is 0:734, while we achieve a ROC AUC of 0.764 when comparing the predictions against OMIM's gene–disease associations and 0.798 using MGI's gene–disease associations as positive instances. The resulting ROC curves, including the updated ROC curves of PhenomeNet when using OMIM's disease phenotypes, are shown in figure 1.

While the resulting ROC curves and their ROC AUC demonstrate the feasibility of our approach, our choice of treating unknown gene–disease associations as negative instances in the evaluation means that these results are conservative estimates of the true performance of our method. Our aim is to find causal genes for orphan diseases without known molecular basis, and in our evaluation, we will treat these as negative instances even if a biological relation exists between the gene and the disease.

### 2.2. *HIP1* as a candidate gene for Bassoe syndrome

The PhenomeNet approach, in contrast to ‘guilt-by-association’ approaches [23], does not require prior knowledge of the genetic basis of diseases for its predictions and is, therefore, ideally suited for investigating diseases whose genetic basis is unknown. We manually investigated the PhenomeNet predictions for Orphanet's diseases and identified *HIP1* as a candidate for the orphan disease *Bassoe syndrome* (ORPHANET:1875, OMIM:254000). An overview of the similarity between the phenotypes of Bassoe syndrome and *HIP1* mutations is illustrated in table 2.

**Table 2.** The phenotypic traits of Bassoe syndrome in Orphanet and the phenotypic manifestations of mutations in *Hip1* available in the MGI database. The last column lists additional phenotypes associated with *Hip1* mutations in mouse found in the scientific literature.

organ system	Orphanet	mouse models (MGI)	additional mouse phenotypes reported in literature
skeletal	kyphosis, hypertensible joints, cubitus valgus	abnormal spine curvature, lordosis	kyphosis [24], kypholordosis [25], spinal defects [26]
muscular	amyotrophy, hypotonia, muscle hypotrophy	abnormal muscle morphology	muscle hypotrophy [27], muscle wasting [27]
behavioural	abnormal gait, amimia	abnormal gait, hypoactivity, tremors	failure to thrive [25], ataxia [24], defects in presynaptic function [27]
visual	cataract, strabismus	nuclear cataracts, microphthalmia	cataracts [26]
reproductive	testicular atrophy, hypogonadism, hypogenitalism, abnormal ovaries, reduced fertility	testicular atrophy, male infertility	decreased testicular weight [28], testicular degeneration [26,28], increased apoptosis of postmeiotic spermatids [28], oligospermia [28], decreased fertility [26,29], reduced sperm count and motility [26,29], ovarian abnormalities [29]

Bassoe syndrome (congenital muscular dystrophy—infantile cataract— hypogonadism) was first described in an extended kindred in Norway with seven affected individuals in four generations and a history of male and female stillbirths [30]. The complexity and severity of the phenotype was very variable but characteristically associated with hypogonadism/gonadal dysgenesis, in one case with elevated gonadotrophins, muscular dystrophy/amyotonia and infantile cataract. Orphanet associates Bassoe syndrome with kyphosis, cataract, hypotonia, muscle hypotrophy, hypogonadism, hypogenitalism, abnormal gait, abnormal ovaries, amimia, amyotrophy, hypoplastic testis, reduced fertility, hyperextensible joints, cubitus valgus and strabismus. The availability of this richer characterization of the syndrome, in contrast to the minimal phenotype-related annotations provided in the OMIM description, allowed our extended version of PhenomeNet to rank the disease as possessing the most similar set of phenotypes to those reported for mutations in the orthologous *Hip1* gene in the mouse and other model organisms. The similarity between the affected individuals in this family to mice carrying null alleles for *Hip1* is striking.

*HIP1* encodes the Huntingtin-interacting protein 1 (HIP1), which has been identified as an interacting partner of Huntingtin, a protein associated with neurodegeneration in Huntington disease. It is expressed in many tissues throughout the body [31] and in different brain regions [32], it has been shown to be involved in clathrin-mediated endocytosis of cell surface receptors [27,33] and it plays a role in development [26] and tumourigenesis [34]. More recently, HIP1 has been implicated in androgen and oestrogen-mediated transcriptional activation, and it has been suggested that it may associate with other promoters or response elements and regulate the transcriptional activity of other nuclear hormone nuclear receptors [35]. Expression of *HIP1* in postmeiotic spermatids reinforces a potential role for germ cell differentiation or maintenance, which is consistent with the mouse phenotypes described to date.

Experimental evidence in mice links *Hip1* mutations to cataracts [26], spinal defects [26], kyphosis [24] and kypholordosis [25], microphthalmia [26], failure to thrive [25] as well as tremors, abnormal gait and ataxia [24]. *Hip1*-null mice were also linked to decreased testicular weight owing to testicular degeneration and increased apoptosis of postmeiotic spermatids and oligospermia [28], decreased fertility, reduced sperm count, and motility and ovarian abnormalities [24,26,29]. *Hip1*-null mice also present complex development-related phenotypes, abnormal hematopoiesis and muscle hypotrophy/wasting [27]. There is debate as to whether the abnormal gait and muscle wasting observed in *Hip1*-null mice are of neurological origin [24,26]. However, *Hip1*-null mice have defects in presynaptic function, delayed recovery from chemically induced long-term depression and altered AMPA and NMDA receptor function [24,27]. The variable severity and expressivity of the *Hip1* alleles made to date, mainly on recombinant congenic backgrounds, suggests that the phenotypes are subject to either background effects or intrinsic threshold variability, with a pattern strongly reminiscent of the family described by Bassoe [30].

More recently, Bradley et al. [36] created a double knockout of *Hip1* and *Hip1r*, the *Hip1-related* protein, with much more severe and penetrant phenotypes such as extreme kyphosis. The protein HIP1r is important in the development of the gastric mucosa [37], providing a possible explanation for the comment from Bassoe in 1956 that his patients suffered from ‘indigestion’ sufficiently severe to merit clinical intervention, if the two have overlapping functionality as suggested by the complementation study conducted by Bradley *et al*. [36].

### 2.3. Human mutations in *HIP1*

With the exception of a fusion protein between *HIP1* and *PDGFR* being recorded as part of a chromosomal translocation in chronic myeloid leukaemia [38], coding sequence or regulatory mutations in *HIP1* have not been reported in humans. In a study of recurrent distal 7q11.23 deletions, statistical analysis of the association between epilepsy and *HIP1* deletion in 10 families with deletions covering the *HIP1* locus showed a significant association [39]. The authors concluded that haploinsufficiency of *HIP1* is sufficient to predispose the brain to epilepsy and a broad range of cognitive and neurobehavioural abnormalities, including intellectual disabilities, hyperactivity, and aggression [39]. This study also reported two reciprocal microduplications inclusive of *HIP1* with behavioural phenotypes related to expressive language disorder, attention deficit hyperactivity disorder and aggression phenotypes, bipolar disorder and encephalocele. This suggests that overexpression may be associated with a similar phenotype as underexpression, and in some cases where the copy number variation (CNV) region was inherited from an unaffected parent there was a suggestion of a two-hit mechanism where a second somatic mutation results in expression of the phenotype. To date, non-neurological phenotypes have not been reported for patients with CNVs including *HIP1* and the phenotype associated with smallest deletion including *HIP1* reported by Ramocki *et al.* [39] is only reported as epilepsy. A recent report of a patient with a chromosome 12q24.31–q24.33 deletion showing developmental delay, kyphoscoliosis and micropenis suggests that loss of *HIP1R* results in a phenotype related to the mouse mutant [40].

The discrepancy between the human and mouse phenotypes for *Hip1*/*HIP1* lesions may be due to ascertainment; Ramocki *et al.* [39] used a database of CNVs to identify patients. In humans, coding sequence or regulatory mutations may be necessary to show the complete phenotype, predicting that patients with Bassoe syndrome might show specific gain-of-function or change-of-function mutations, or may be functionally null rather than haploinsufficient; heterozygous knock-out mice show weaker phenotypes in comparison with complete nulls [24]. The demonstration that human *HIP1* can almost completely compensate for removal of *Hip1* and *Hip1r* strongly suggests that the two genes are functionally equivalent in mouse and human [36].

## 3. Material and methods

### 3.1. Ontology-based cross-species integration

To make phenotypes of animal models comparable with human phenotypes, we follow a knowledge-based approach using biomedical ontologies and automated reasoning. Phenotypes, clinical signs and symptoms are widely represented using biomedical ontologies, such as the Human Phenotype Ontology (HPO) [4] and the Mammalian Phenotype Ontology (MP) [5]. Many phenotype ontologies used in model organisms and humans have been defined based on the PATO framework [18,19]. In these definitions, phenotypes, signs and symptoms are decomposed in an affected *entity* and a *quality* that characterizes how the entity is affected. Entities in phenotypes, clinical signs and symptoms are either *biological processes* and *functions* or *anatomical structures*. Processes and functions, such as *mating* (GO:0007618), are represented using the species-independent GO [41], whereas anatomical entities are commonly represented using species-specific anatomy ontologies.

Phenotypes in which functions and processes are affected are directly comparable between species owing to the use of the species-independent GO and the species-independent PATO ontology. To make phenotypes in which anatomical structures are affected comparable between species, *homologous* anatomical structures between species can be identified and used to systematically integrate phenotypes across species [42]. To account for gaps between species, as well as different levels of granularity in anatomy ontologies, background knowledge in ontologies can be used to provide an additional layer of abstraction. For example, we can compare the human phenotype *Proximal fibular overgrowth* (HP:0005067, decomposed into the entity *Proximal epiphysis of fibula* (human) and the quality *Hypertrophic*) and the mouse phenotype *Abnormal fibula morphology* (MP:0002187, decomposed into the entity *Fibula* (mouse) and the quality *Abnormal morphology*). For this purpose, we make use of the knowledge that *Fibula* (mouse) and *Fibula* (human) are homologous anatomical structures, that *Proximal epiphysis of fibula* (human) is a part of *Fibula* (human), and that *Hypertrophic* is a kind of *Abnormal morphology*. We then infer, using automated reasoning, that *Proximal fibular overgrowth* (human) is a kind of *Abnormal fibula morphology* (mouse). In PhenomeNet, we formalize EQ-based phenotype definitions in the Web Ontology Language (OWL) [43] and use the consequence-based OWL reasoner CB [44] to infer related phenotypes across species. The source code and the resulting mappings are freely available at http://phenomeblast.googlecode.com.

### 3.2. Semantic similarity

To analyse information from phenotype ontologies and compare phenotypic similarity between animal models, diseases and drug profiles, we use a measure of semantic similarity [45]. Semantic similarity exploits the background knowledge in an ontology, commonly the ontology's underlying graph structure, to identify similar concepts. In particular, we use the simGIC similarity measure [46]. simGIC is based on the Jaccard metric, which is a measure to compare set similarity, and can be used to evaluate the distance between two sets of phenotype terms. To make the Jaccard metric a *semantic* similarity measure between a set of phenotype terms *S*_1_ and another set of phenotype terms *S*_2_, using the ontology *O* as background knowledge, simGIC adds, for every element *x* of *S*_1_ and *y* of *S*_2_, the superclasses of *x* in *O* to *S*_1_ and the superclasses of *y* in *O* to *S*_2_ (i.e. it compares sets that are closed against the super-class relation). To compare the similarity between two diseases, we then calculate the information content *I*(*x*) of each phenotype term *x* in our integrated phenotype resource. The information content *I*(*x*) of the term *x* is defined based on the probability *P*(*X* = *x*) that a gene or disease is characterized with *x*3.1
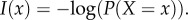


We then calculate the similarity between the sets *S*_1_ and *S*_2_ (closed against the super-class relation) as3.2
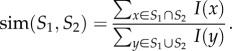


The use of semantic similarity has several benefits over other similarity measures. It benefits from the background knowledge in ontologies, in particular the hierarchical abstraction that ontologies provide, and can define similarity based on overlap of general features of a phenotype (e.g. a similarity between the anatomical location affected in a phenotype instead of an exact match). In PhenomeNet, we close sets of phenotype terms against superclasses in the MP, because the use of MP has been shown to yield the best results when analysing mouse phenotypes [47].

### 3.3. Mapping of Orphanet clinical signs to Human and Mammalian Phenotype Ontology

We have created a phenotypic representation of the disorders in Orphanet based on the HPO and MP [4,5]. To generate the mapping between Orphanet's clinical signs, and HPO and MP terms, we used a combination of lexical, structural and manual approaches. First, we use the Needleman–Wunsch algorithm [48] to find the labels and synonyms of phenotype terms in the HPO and MP that are lexically most similar to the labels of clinical signs in Orphanet, and we assign these MP or HPO classes as *equivalent* to the clinical sign in Orphanet. Second, we use the taxonomic structure of clinical signs in Orphanet and identify a *superclass* in HPO or MP for clinical signs. In particular, we identify a superclass, in Orphanet's classification of clinical signs, which is lexically identical or very similar to a term in the HPO or MP, and assign this HPO or MP term as a superclass of Orphanet's clinical sign. Finally, we manually reviewed the mappings and removed incorrect associations. As a result, we can associate 2507 disorders from OrphaNet with 52 002 terms from HPO as well as 11 674 phenotype terms from MP.
